# Formation mechanism of carbide slag composite sustained-alkalinity-release particles for the source control of acid mine drainage

**DOI:** 10.1038/s41598-021-03277-w

**Published:** 2021-12-10

**Authors:** Jichi Bai, Haiqin Zhang, Liping Xiao

**Affiliations:** 1 School of Mines, Liaoning Technical University, Fuxin, 123000 China; 2 College of Environmental Science and Engineer, Liaoning Technical University, Fuxin, 123000 China; 3 School of Environmental and Municipal Engineering, Qingdao University of Technology, Qingdao, 266525 China

**Keywords:** Pollution remediation, Composites

## Abstract

Acid mine drainage (AMD) has caused serious and long-lasting damage to the environment in many countries. Preventing AMD formation at the source is considered the most direct and effective method of remediation. Carbide slag, an industrial waste, is a potential AMD treatment material due to its strong alkalinity. However, applying carbide slag at the source carries difficulties due to its rapid release of alkalinity. This is the first attempt to mix carbide slag with bentonite to prepare sustained-alkalinity-release particles for source control of AMD. The size of Ca(OH)_2_ crystallites is decreased from 267 to 211 nm, and the reduced part forms calcium silicate hydrate gel (C–S–H) between the carbide slag and bentonite. C–S–H encapsulated on the surface of the carbide slag, increasing the mechanical strength of the particles, and achieving slow release of alkalinity. The suggested optimum preparation conditions for the particles are as follows: bentonite-to-carbide slag mass ratio of 3:7, Na_2_CO_3_ dose of 10 wt%, and calcination temperature of 500 °C for 1 h. The particles can remove 105 mg/g Cu^2+^ within 12 h, and the loss rate is only 7.4%. The alkalinity release time of the particles is 4 times greater than that of carbide slag.

## Introduction

Mineral resources are a perpetual driving force of social development, but the environmental problems caused by mining have become increasingly prominent and persist for a long time, even after mine closure. Pollutants in mining areas diffuse to the atmosphere^1,2^, soil^3,4^ and rivers^5^. Acid mine drainage (AMD) is characterized by its concealment, strong acidity, high concentrations of heavy metals, and persistence^6,7^. The Picher mining area in the United States^8^ and the Carnoulès mine in France^9^ are typical cases of serious AMD pollution. AMD has caused serious damage to approximately 72,000 hectares of lakes and reservoirs around the world and has become a serious environmental problem worldwide^10^.

The formation of AMD is due to the combined action of microorganisms and oxygen, as well as sulfide oxidation during the cyclic transformation of Fe^2+^ and Fe^3+^. The methods of blocking one or two formation conditions at the source to reduce the risk of AMD formation are usually called “source control” measures. Compared with “migration control” measures, “source control” minimizes the formation and migration of AMD^11^; the relevant technologies include inhibition of live bacteria^12^, alkaline material filling^13,14^, waste packaging^15,16^, wetland construction^17,18^ and anoxic limestone drains (ALDs)^19^. ALDs were first proposed by Turner and McCoy in 1990^20^. This process has low requirements on the topography and raw water quality of the mining area and has been considered the best source control method. ALDs can prevent limestone from undergoing “armouring” under anaerobic conditions. The alkalinity released by limestone can increase the pH value of AMD while removing heavy metal ions. However, limestone can only increase the pH to 8 and cannot completely remove metal ions such as Fe^2+^ and Mn^2+^^21^. Moreover, FeCO_3_ and MnCO_3_ colloids are easily generated and thus cause incongruent dissolution, which shortens the service life of the structure^22^. Therefore, overcoming the above shortcomings and developing the alternative limestone materials have become the goals of many studies.

Blast furnace slag^23^, fly ash^24^ and serpentinite^25,26^ have all been considered as replacements for limestone. Petrik^27^ suggested that fly ash alone is a cost-effective alkaline treatment material, but fly ash increases the effluent turbidity and is not conducive to the separation of metals and raw materials. Turigan^28^ replaced limestone with low-grade nickel soil, but the nickel soil could buffer the pH to only 4.7 at the highest, allowing very few types of heavy metals to be removed. The main component of carbide slag is Ca(OH)_2_, which is highly soluble, releases a large amount of alkalinity and has a high removal efficiency for heavy metal ions^29^. Based on the above advantages, carbide slag has been used for fixation of heavy metals in soil and removal of phosphorus from wastewater in recent years^30,31^. However, there are few studies on the treatment of AMD with carbide slag instead of limestone. Due to the fast alkalinity release rate of carbide slag, the service life may be shortened. Therefore, it is necessary to modify and reduce the alkalinity release rate. In the presence of silicate or a more aluminosilicate-rich precursor, such as calcium silicate or fly ash, Ca(OH)_2_ reacts via the pozzolanic reaction to produce cementitious products such as calcium silicate hydrate (C–S–H), calcium aluminate hydrate (C–A–H) and calcium aluminate silicate hydrates (C–A–S–H), which improves the mechanical strength of carbide slag^30,32^. Sun found that the formation of hydration products could slow the alkalinity release rate of Ca(OH)_2_, but an excessive gel content led to a decrease in permeability after hardening^30,33^. Therefore, a clay mineral with high permeability, bentonite, may be the best choice for addressing the above-mentioned defects.

In this study, carbide slag was used as the main material, bentonite was used as the Si/Al source, and Na_2_CO_3_ was used as the activator to granulate the two kinds of powder material to prepare sustained-alkalinity-release particles for ALDs. It is expected that the hydration products generated by the reaction between the raw materials will enhance the particle strength and the slow release of alkalinity. The effects of preparation factors such as the ratios of bentonite to calcium carbide slag, the Na_2_CO_3_ dose and the calcination temperature were analysed. Cu^2+^, which is one of the most concerning heavy metal ions (Cu^2+^, Pb^2+^, Zn^2+^ et al.) in AMD^34^, was selected to evaluate the removal of heavy metals by the particles. The loss rate was measured to evaluate the mechanical strength of the particles. The effects of various factors on the particle composition and microstructure were characterized by X-ray diffraction (XRD) and scanning electron microscopy (SEM). The optimum preparation conditions for the carbide slag composite sustained-alkalinity-release particles were determined, and the alkalinity release rates of the particles and carbide slag were compared.

## Materials and methods

### Materials and chemicals

The carbide slag used in this study was provided by Fuxin Acetylene Factory, Liaoning Province, China, and the bentonite was provided by Wanpeng Mine, Liaoning Province, China. The X-ray fluorescence (XRF) results used to determine the composition of the two materials are shown in Table 1. Before the experiment, the two raw materials were ground, passed through a 200-mesh screen, and dried in an oven at 105 °C for 2 h.

**Table 1:** Chemical compositions of the carbide slag and bentonite used, in oxide %wt.

Oxide	CaO	SiO_2_	Al_2_O_3_	Na_2_O	Fe_2_O_3_	MgO	TiO_2_	K_2_O
Carbide slag	93.65	2.97	0.82	0.71	0.22	0.15	0.06	-
Bentonite	2.24	73.33	13.55	4.25	2.26	3.59	0.12	0.50

Small amounts of Na, Al and other metal ions in the carbide slag were detected (Table 1); these ions may have been derived from the acetylene raw material (calcium carbide) or mixed impurities in the production process. According to the chemical composition of the bentonite, a large amount of Na^+^ replaced Ca^2+^, which belonged to Na-bentonite. Small amounts of iron, magnesium, potassium and titanium were also detected (Table 1), which may have been caused by substitution of the aluminium oxide octahedral structures in montmorillonite (MMT) by other divalent cations^35^. The XRD results are shown in Fig. S1, the main mineral components of bentonite were MMT and SiO_2_, and the main component of the carbide slag was Ca(OH)_2_.

Cu^2+^ was used in the adsorption experiment. The copper solution was prepared from CuSO_4_·5H_2_O. HNO_3_ and NaOH were used to adjust the pH of the solution. Na_2_CO_3_ was used as an activator for the preparation of the particles. The above chemical reagents were analytically pure.

### Preparation of the granular composites

The preparation of composite particles mainly includes raw material mixing, particle forming and high-temperature roasting. First, bentonite and carbide slag were mixed according to mass ratios of 1:9–9:1. Additionally, to study the effect of Na_2_CO_3_ on the particles, 0–10 wt% Na_2_CO_3_ was added to the raw materials. To ensure the raw materials met the plastic requirements of machine processing, distilled water was added to the raw materials, and the moisture content was controlled at 60%. The raw materials and water were mixed by hand for 15 min to ensure the full dispersion and dissolution of the mixture. Then, the mixture was machine processed into small cylindrical particles with a diameter of 2 mm and a length of 3 mm. Finally, the formed particles were calcined at 200–800 °C and then cooled to room temperature. In order to compare the influence of calcination temperature on the particles, uncalcined particles were prepared at room temperature and stored in sealable plastic bags. In the following discussion, the particles obtained under different conditions were named with the mass ratio of bentonite to carbide slag.

### Copper removal experiment

A series of particles were prepared according to different mass ratios of bentonite to carbide slag, doses of Na_2_CO_3_, and calcination temperatures. By comparing the removal rates of Cu^2+^, the treatment effect of the particles on copper-containing wastewater was analysed. The pH was adjusted to 3.2 (± 0.05) with 10% HNO_3_ and 10% NaOH. Briefly, 0.20 g composite particles were placed into a 250 mL beaker, and 100 mL simulated wastewater at a concentration of 300 mg/L was added. The solution was treated for 12 h under static conditions. The residual Cu^2+^ concentration in the solution was determined by flame atomic spectrophotometer (HITACHI Z-2000, Japan) at a wavelength of 324.8 nm.

### Characterization of granular composites

The mass loss of particles during wastewater treatment is mainly caused by hydraulic shear, collision and friction between particles and/or between particles and container walls^36^. Therefore, the loss rate was used to characterize the mechanical strength of the particles, which to some extent match the breakage mechanisms in practical use of wastewater treatment^37,38^. The specific operation involved adding a consistent mass of particles to a 250 mL conical flask, adding 100 mL distilled water, and placing the flask in a shaker at 100 rpm for 12 h. Then, the particles were dried at 105 °C for 2 h, cooled at room temperature and passed through a 0.5 mm mesh sieve. The remaining particles were weighed, and the loss rate was calculated as follows^38^:

$$\rho =\frac{{m}_{1}-{m}_{2}}{{m}_{1}}\times 100\%$$

where *ρ* is the loss rate of composite particles (%), *m*_1_ is the initial mass of composite particles (g), and *m*_2_ is the mass of the composite particles after drying (g). The larger the loss rate is, the worse the mechanical strength of the particle.

The morphology and structure of the different particles were characterized by SEM (German Nova 400 Nano). The accelerating voltage was 10 kV. Due to the low conductivity of the composite particles, the surface of the particles needed to be sprayed with gold for 3 min before testing.

The mineral composition of the particles was characterized by XRD (Rigaku Ultima IV, Japan). The particles were manually ground into powder and tested by X-ray bombardment with a Cu-Kα radiation source. The specific parameter settings were as follows: the incident wavelength was λ = 1.5418 Å, the power supply voltage was 40 kV, the current was 40 mA, the scanning angle was 5°–90°, and the angular velocity was 4°/min.

## Results and discussion

### Effect of the mass ratio of bentonite to carbide slag on the particles

**Figure 1:**
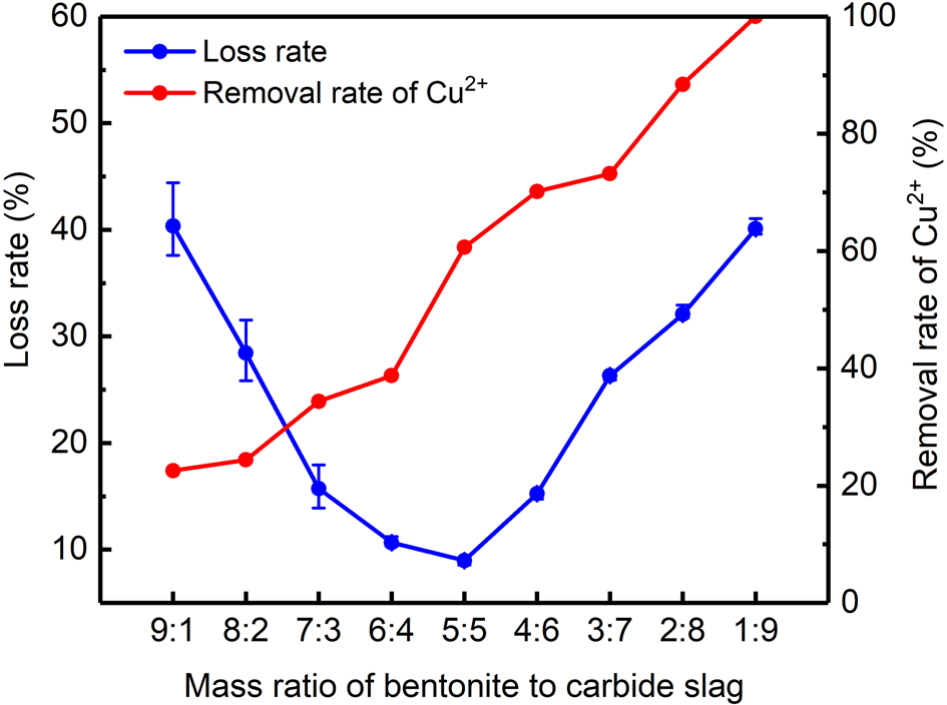
Loss rate and Cu^2+^ removal rate for particles with different mass ratios of bentonite to carbide slag.

Bentonite and carbide slag were mixed at mass ratios of 9:1–1:9, and 5 wt% Na_2_CO_3_ was added. Then, the mixture was roasted at 500 °C for 1 h to obtain a series of particles. The removal rate of Cu^2+^ and loss rate of particles prepared with different mass ratios of bentonite to calcium carbide are shown in Fig. 1. Increasing the proportion of carbide slag was conducive to the formation of Cu^2+^ precipitates. When the ratio of bentonite to carbide slag decreased from 9:1 to 5:5, the particle loss rate decreased from 40.36 to 8.98%. However, when the ratio decreased to 1:9, the particle loss rate increased to 40.1%. This indicates that the loss rate could be controlled by changing the ratio.

**Figure 2:**
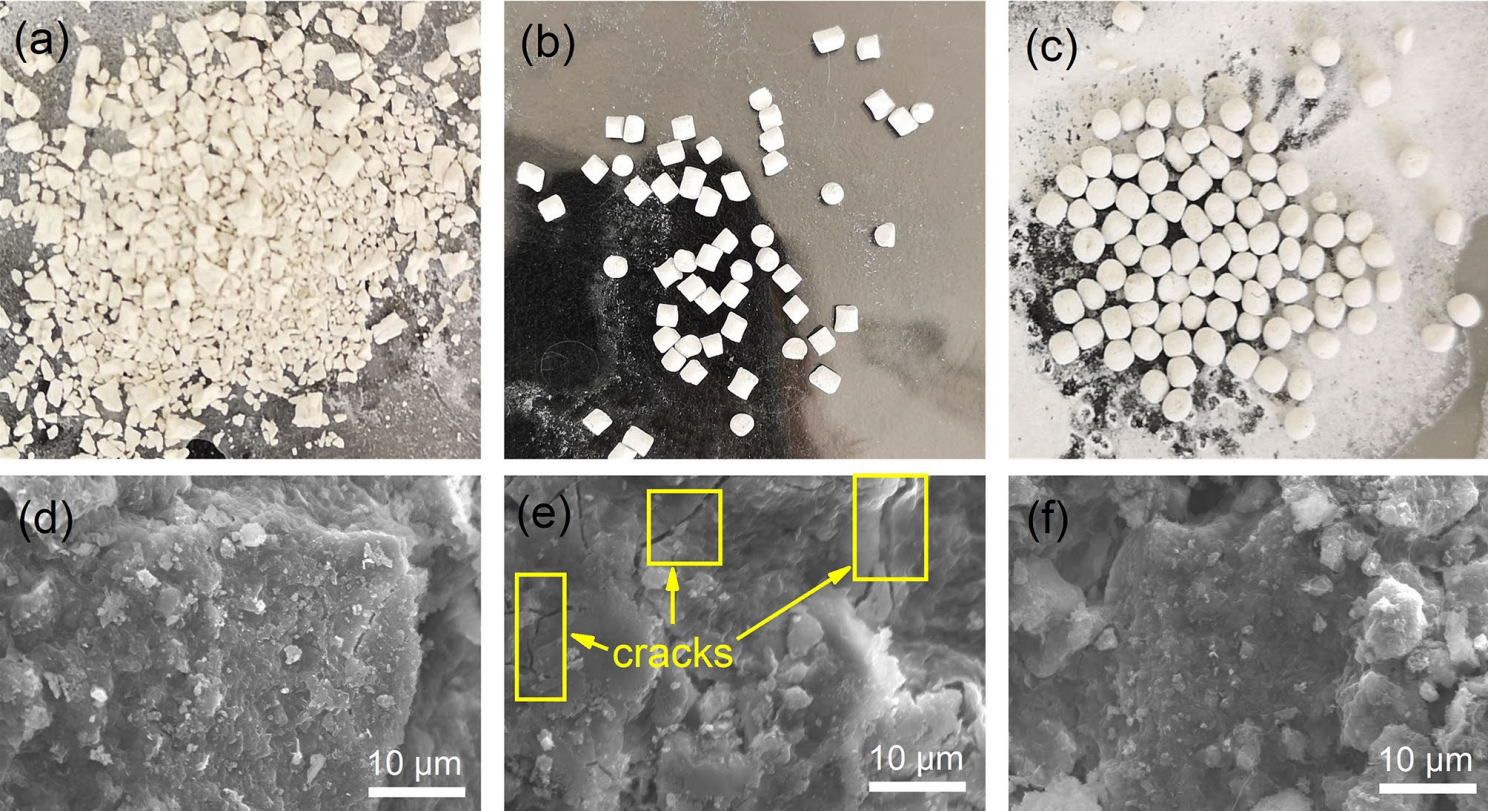
(**a**–**c**) Appearance after loss rate experiments with typical particles and (**d**–**f**) SEM images of typical particles: (**a**), (**d**) 9:1 particles, (**b**), (**e**) 5:5 particles, and (**c**), (**f**) 1:9 particles.

The losses of three typical particles in water are shown in Fig. 2a–c. As the contact time with water increased, the 9:1 particles gradually ruptured into small blocks, while the 1:9 particles gradually became spherical. These morphological changes indicate that when the ratio of bentonite to carbide slag is greater than 5:5, internal force is caused by bentonite swelling due to water absorption^39^. When the ratio is less than 5:5, external forces such as flow shear and friction are concentrated at the corners of the particles, leading to the separation of powder from the particle surface. This separation is the main reason for the increase in loss rate, indicating that the internal cohesion of the particles is insufficient. The particle morphology is complete when the ratio of bentonite to carbide slag is 5:5, indicating that the cohesive force inside particles can overcome the expansion of bentonite, water shear and friction to achieve balance. Thus, the loss rate reaches a minimum.

The surface morphologies of the three typical particles are compared in Fig. 2d–f. As shown in Fig. 2d, the 9:1 particle surface exhibits a smooth, crack-free compact structure. Figure 2e corresponds to the 5:5 particles; small obvious cracks are observed on their surface. These cracks provide buffer space for bentonite to absorb water and expand, thereby reducing the particle loss rate. The surface morphology of the 1:9 particles is shown in Fig. 2f. The surface is rough, and the number of cracks is decreased. This structure shows that particles exposed on the surface more easily fall off in water under the action of shear and friction, as shown by the macro-morphology in Fig. 2c.

**Figure 3:**
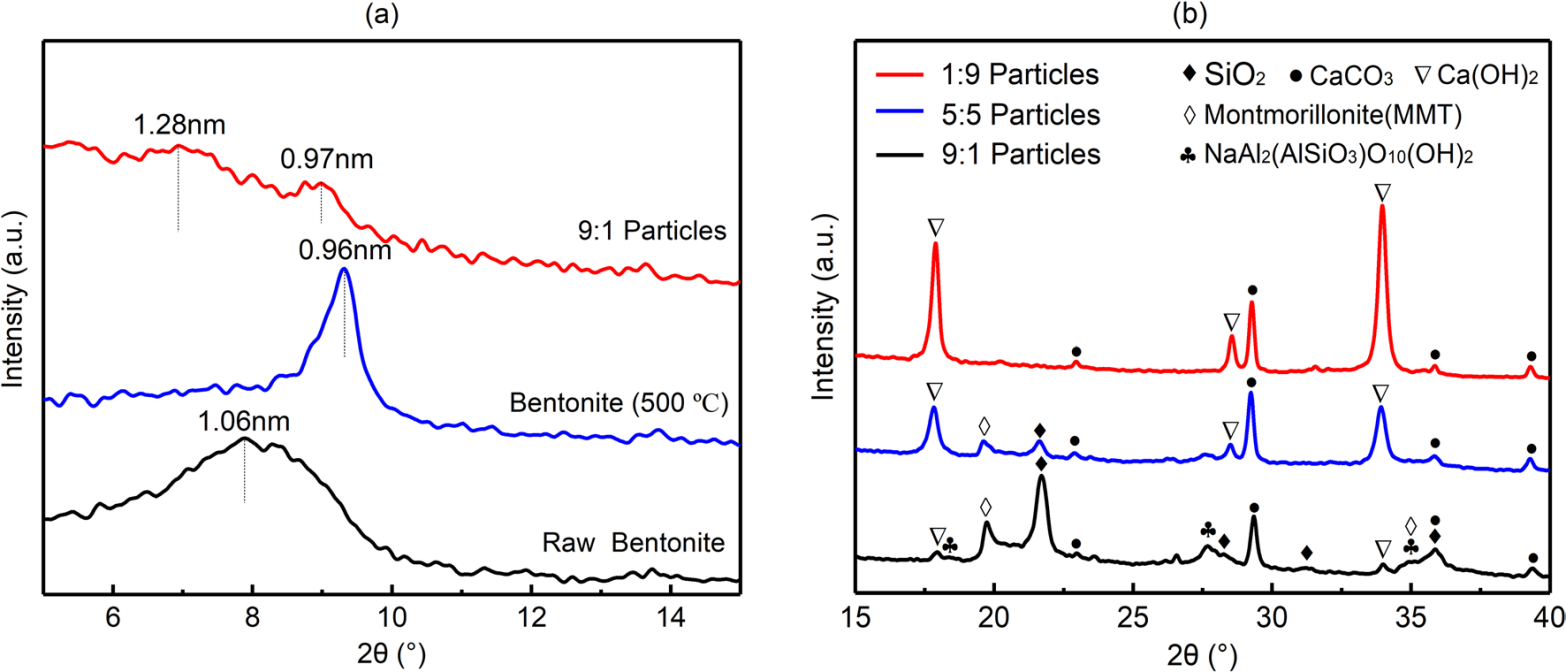
XRD diffraction data: (**a**) uncalcined bentonite, bentonite calcined at 500 °C and 9:1 particles, (**b**) 9:1 particles, 5:5 particles and 1:9 particles.

The changes in the interlayer spacing of montmorillonite in unburned bentonite, calcined bentonite (500 °C) and 9:1 particles are shown in Fig. 3a. No characteristic peaks representing interlayer spacing were found in the 5:5 and 1:9 particles, indicating that the layered structure disappeared. The d_(001)_ value of raw bentonite was 1.06 nm, which is the characteristic peak of sodium montmorillonite. After calcination at 500 °C, the d_(001)_ value decreased to 0.96 nm. This result is consistent with the interlayer spacing of montmorillonite without interlayer-bound water. However, when a small amount of carbide slag was added, there were two weak characteristic peaks within 2θ = 6°–10°. The d_(001)_ value of the characteristic peak of the layered structure increased to 1.28 nm. This result is consistent with the d_(001)_ value of Ca-montmorillonite containing a layer of crystal water^36^, indicating that Ca^2+^ in carbide slag enters the interlayer of montmorillonite, resulting in the transformation of some Na-bentonite to Ca-bentonite. The d_(001)_ value of another characteristic peak was consistent with that of montmorillonite which had lost its interlayer-bound water. This part of bentonite is the main cause of the water swelling and cracking of particles.

Figure 3b compares the phase change of particles at different ratios. The 9:1 particles show an obvious increase between 18° and 25°, indicating that new crystalline phases were formed, but their crystallinity was low. The characteristic SiO_2_ peaks at 31.28° and 35.89° widened, and a flat and wide peak was observed at 27.79°. Phase analysis showed that SiO_2_ was transformed into NaAl_2_(AlSiO_3_)O_10_(OH)_2_, indicating that the crystallinity of SiO_2_ decreased and silicate was formed. As the ratio was decreased to 5:5, the characteristic peaks of SiO_2_ at 31.28° and 35.89° disappeared. No new peaks appeared, indicating that SiO_2_ existed in an amorphous form. When the ratio was further decreased to 1:9, the characteristic peak of montmorillonite at 19.88° disappeared, indicating that montmorillonite was transformed into an amorphous state.

The above results indicate that the formation of an amorphous gel is conducive to enhancing the mechanical strength of particles and SiO_2_ in bentonite is preferentially converted to an amorphous state over montmorillonite. Therefore, when the proportion of bentonite is greater than 5:5, the layered structure of montmorillonite is retained, and the particles are more likely to be destroyed due to water absorption and expansion. When the proportion of bentonite is less than 5:5, the insufficient production of gel leads to an increase in the loss rate.

### Effect of Na_2_CO_3_ dose on the particles

It can be seen from “Effect of the mass ratio of bentonite to carbide slag on the particles” that increasing the proportion of carbide slag can effectively improve the removal rate of Cu^2+^ ions, and the formation of an amorphous gel is conducive to enhancing the particle strength. According to Provis et al.^40^, the formation of C–S–H, C–A–H, and C–A–S–H gels depends on the activation of alkali metal hydroxides or carbonates (i.e., Na_2_CO_3_). Increasing the proportion of calcium carbide slag as much as possible while maintaining a low loss rate is desirable. Therefore, a series of particles were obtained by increasing the dose of Na_2_CO_3_ from 0 to 10% at different ratios (5:5, 4:6, 3:7) and roasting at 500 °C for 1 h.

**Figure 4:**
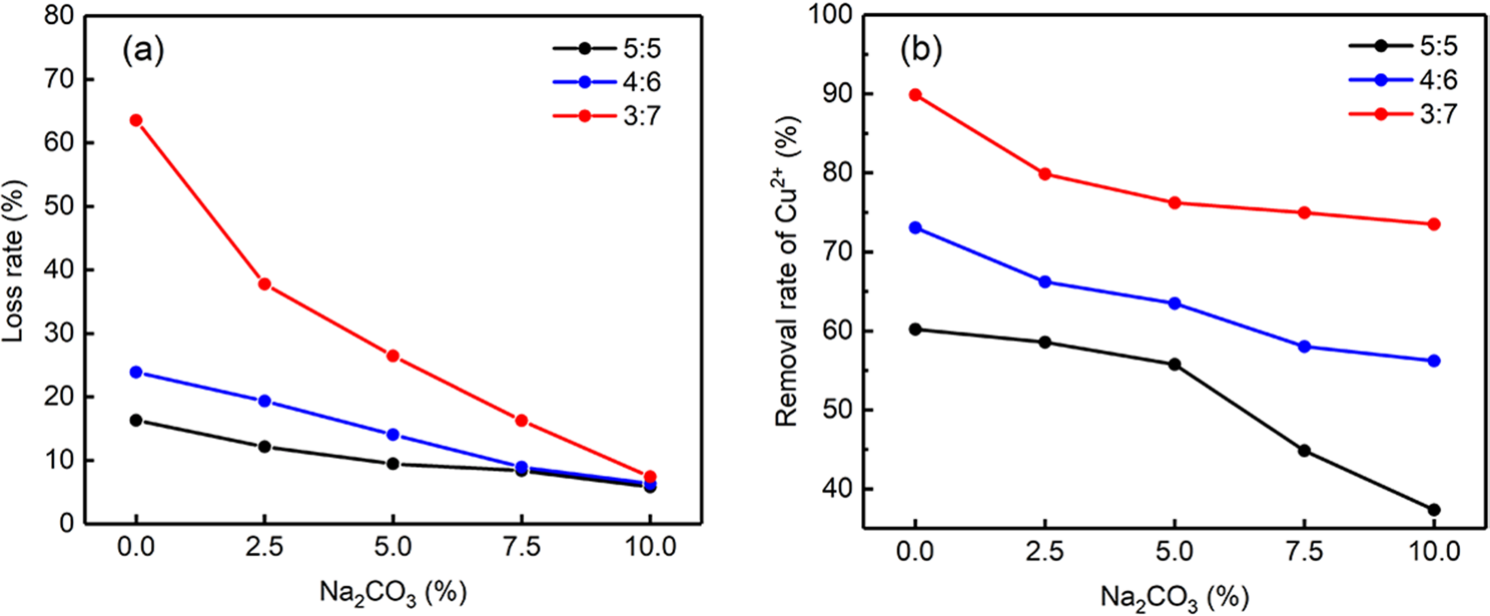
Effect of different Na_2_CO_3_ doses on (**a**) the particle loss rate and (**b**) the Cu^2+^ removal rate.

Figure 4 shows the loss rate and Cu^2+^ removal rate for the different particles. The Na_2_CO_3_ content was increased from 0 to 10 wt%, and the loss rates of the three groups of particles was decreased to different degrees. The loss rate of the 3:7 series of particles decreased the most, from 63.56 to 7.40%. When 10 wt% Na_2_CO_3_ was added, the loss rate of the 3:7 particles (7.40%) was similar to the loss rate (5.82%) of the 5:5 particles (Fig. 4a). These loss rate results indicate that increasing the amount of Na_2_CO_3_ can reduce the loss rate.

The removal rate of Cu^2+^ decreased when increasing the Na_2_CO_3_ dose from 0 wt% to 10 wt% (Fig. 4b), indicating that the addition of Na_2_CO_3_ had a negative effect on the removal of Cu^2+^. However, the removal rate of Cu^2+^ with the 3:7 particles was better than those of the other two groups. The loss rate of the 3:7 particles with 10 wt% Na_2_CO_3_ was less than 10%.

**Figure 5:**
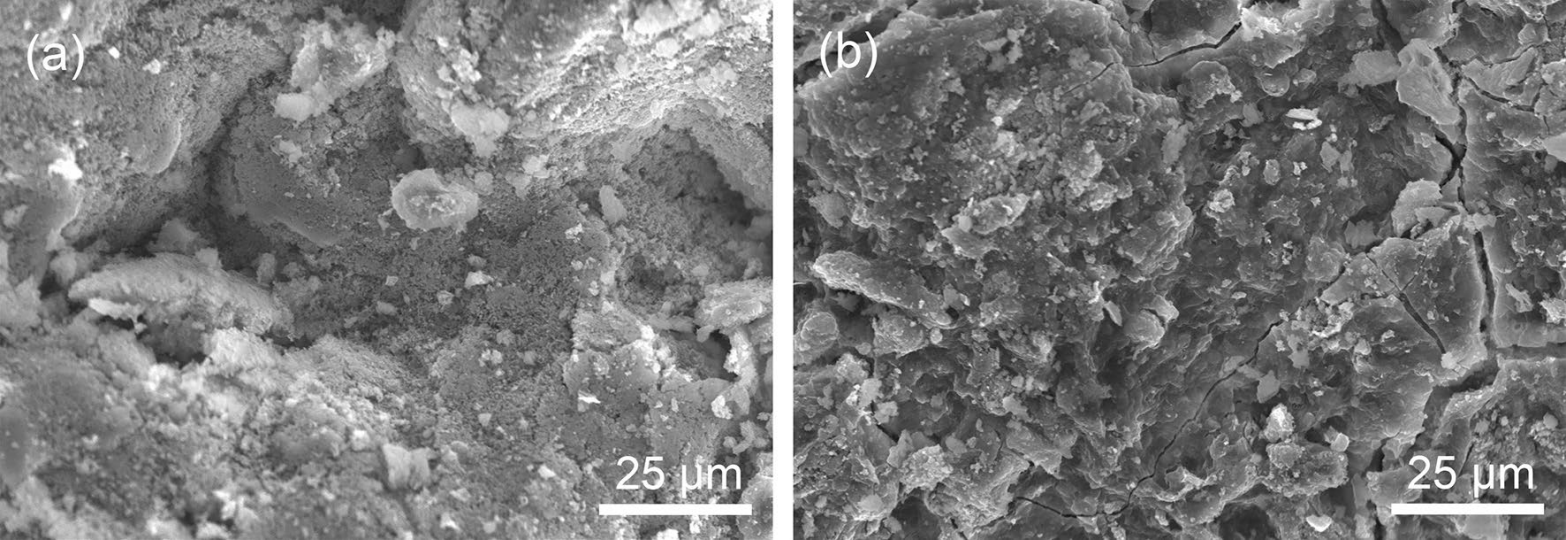
SEM images of 3:7 particles with (**a**) 0% and (**b**) 10% Na_2_CO_3_.

**Figure 6:**
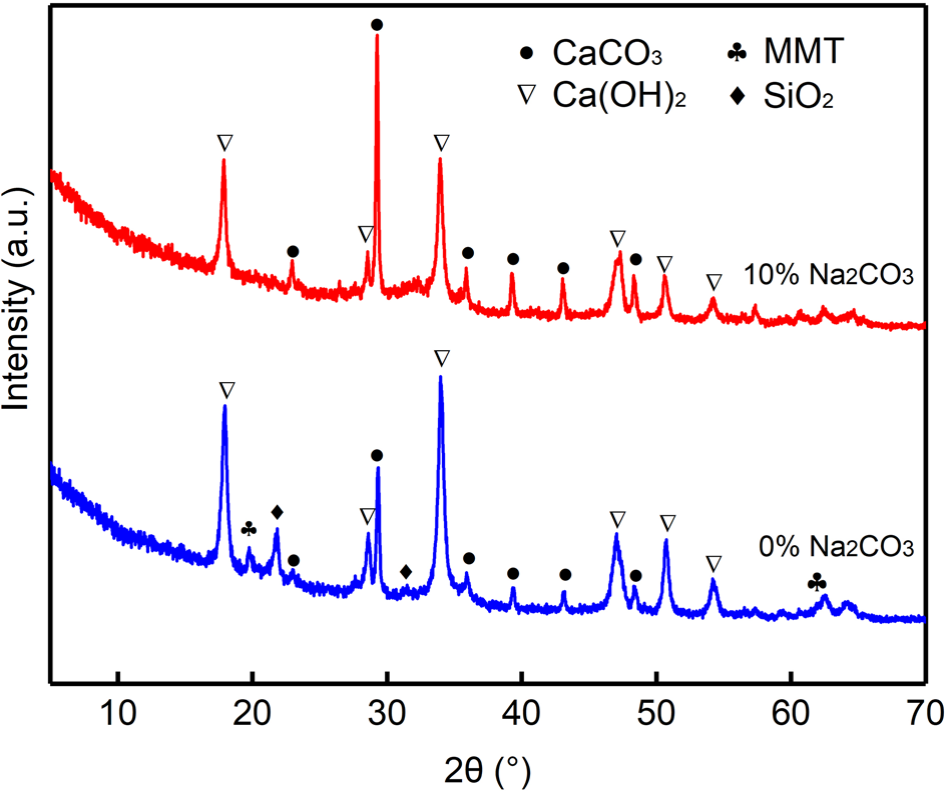
XRD patterns of 3:7 particles without Na_2_CO_3_ and with 10 wt% Na_2_CO_3_.

The surface morphologies of the 3:7 particles with 0 wt% and 10 wt% Na_2_CO_3_ are shown in Fig. 5. The surfaces of the particles made without Na_2_CO_3_ mainly consisted of large irregular particles stacked together in a loose structure, and the edges are rough (Fig. 5a). This structure led to a high loss rate of particles. The XRD analysis of the particles without Na_2_CO_3_ (Fig. 6) shows that the composition of the particles includes montmorillonite and SiO_2_ from the bentonite and Ca(OH)_2_ and CaCO_3_ from the carbide slag. This indicates that the main composition remained unchanged. A slight bulge in the substrate was observed between 25° and 40° because Ca(OH)_2_ generates amorphous C–S–H by pozzolanic reactions^41,42^. However, the amount of amorphous C–S–H was not enough to reduce the loss rate.

When increasing the Na_2_CO_3_ content to 10 wt%, the particle surface flattened, and cracks with clear edges appeared, indicating that the particles lost water and shrank; thus, the structure became compact (Fig. 5b). From Fig. 6, the characteristic peaks of the particles with 10 wt% Na_2_CO_3_ at 19.79° and 21.73° disappeared, and the background hump between 25° and 40° became more obvious than that for the particles without Na_2_CO_3_. This result indicates that Na_2_CO_3_ plays a promoting role in the transformation of montmorillonite and SiO_2_ to an amorphous state because Na_2_CO_3_ can enhance the reactivity of Al and Si in clay and promote the formation of gels^43^. Therefore, with increasing Na_2_CO_3_ dose, the transformation of SiO_2_ and montmorillonite to an amorphous state was promoted, the gel content in the particles increased, and the particle loss rate was effectively reduced.

### Effect of the calcination temperature

**Figure 7:**
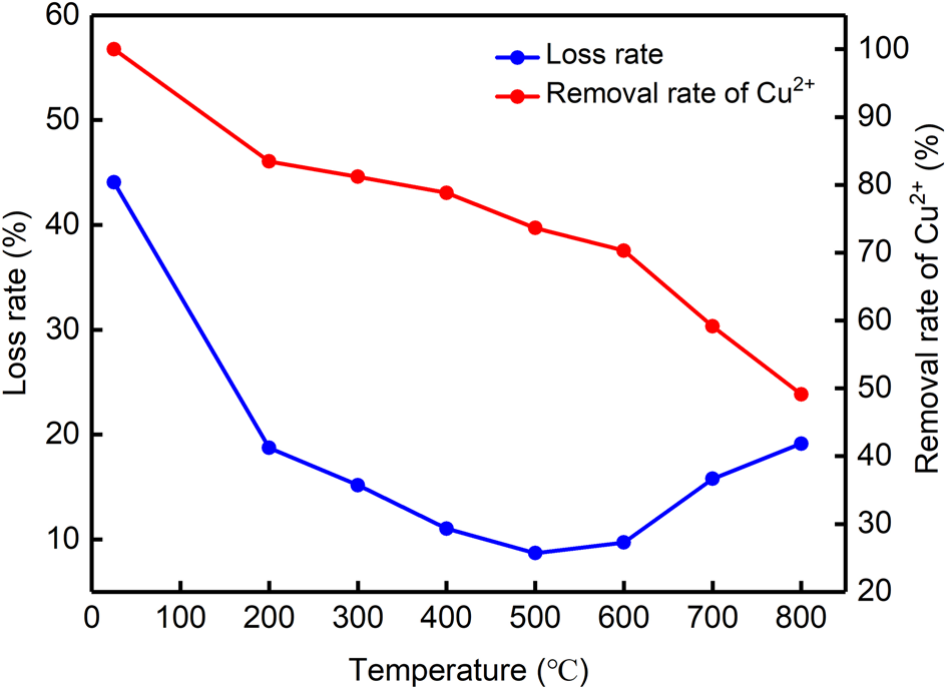
Effect of different calcination temperatures on the loss rate and Cu^2+^ removal rate of 3:7 particles.

When the mass ratio of bentonite to carbide slag was 3:7 and the dose of Na_2_CO_3_ was 10 wt%, a series of particles were obtained after treatment for 1 h at different calcination temperatures to study the effect of calcination temperature on the particles. In the particles prepared at room temperature, the loss rate reached 44.07%, and the removal rate of Cu^2+^ reached 100%. In the particles calcined at 200 °C, the loss rate greatly decreased to 18.73%, and the Cu^2+^ removal rate decreased to 83.46% (Fig. 7). This decrease indicates that the mechanical strength of uncalcined particles is poor, and an excessive loss rate leads to the removal of Cu^2+^, which is consistent with the research results of Zhan^38^. However, an excessive loss rate leads to an increase in effluent turbidity and sludge. When increasing the temperature from 200 to 500 °C, the loss rate of particles decreased from 18.73% to 8.69%. However, when the calcination temperature increased to 800 °C, the loss rate increased to 19.14%. The removal rate of Cu^2+^ decreased with an increasing preparation temperature. According to Fig. 7, the loss rate of particles calcined at 500 °C was the smallest, and the removal ratio of Cu^2+^ was 73.65%. Therefore, 500 °C was set as the preparation temperature of the particles.

**Figure 8:**
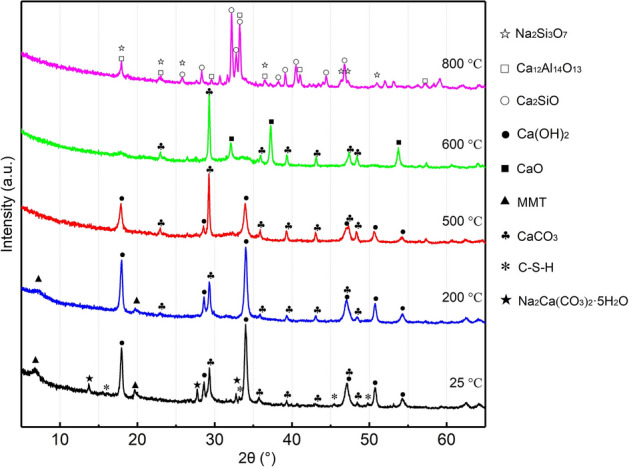
XRD patterns for composite particles at different temperatures.

The XRD patterns of the particles prepared at different temperatures are compared in Fig. 8. In the uncalcined particles, Na_2_Ca(CO_3_)_2_·5H_2_O was detected from characteristic peaks at 2θ = 13.84°, 27.83° and 32.86°, while the characteristic peaks of Na_2_CO_3_ disappeared. This result indicates that a hydration reaction between Na_2_CO_3_ and carbide slag occurs, providing a precursor for the combination of bentonite and carbide slag after calcination. The characteristic peak of SiO_2_ from bentonite disappeared, while the montmorillonite structure was retained. This is because montmorillonite with Al and Si in its crystal structure cannot participate in the pozzolanic reaction^44^. Therefore, SiO_2_ preferentially transforms to an amorphous state over montmorillonite, forming C–S–H and other hydrates. The formation of hydration products gives the particles a certain mechanical strength, but insufficient production leads to a high particle loss rate. As the calcination temperature was increased to 500 °C, the characteristic peaks of montmorillonite at 7.12° and 19.72° disappeared, indicating that the layered structure had collapsed, and the montmorillonite structure was destroyed. This is because the increase in the content of amorphous silicate transferred from Si and Al in the montmorillonite crystal structure with increasing temperature further decreased the loss rate. This is consistent with the research results of Okano^45^.

Calcium hydroxide (CH) can react with aluminosilicates (AS_2_) and water to generate gel products, such as C–S–H, stratlingite (C_2_ASH_8_), tetracalcium aluminate hydrate (C_4_AH_13_) and tricalcium aluminate hexahydrate (C_3_AH_6_). The reaction can be summarized as follows^41,46^:

$${\text{6CH}}\;\left( {\text{s}} \right) + {\text{AS}}_{{2}} \;\left( {\text{s}} \right) + {\text{9H}}\;\left( {\text{l}} \right) \to {\text{C}}_{{4}} {\text{AH}}_{{{13}}} \left( {\text{s}} \right) + {\text{2C}} - {\text{S}} - {\text{H}}\;\left( {\text{s}} \right)$$

$${\text{5CH}}\;\left( {\text{s}} \right) + {\text{AS}}_{{2}} \;\left( {\text{s}} \right) + {\text{3H}}\left( {\text{l}} \right) \to {\text{C}}_{{3}} {\text{AH}}_{{6}} \;\left( {\text{s}} \right) + {\text{2C}} - {\text{S}} - {\text{H}}\;\left( {\text{s}} \right)$$

$${\text{3CH}}\;\left( {\text{s}} \right) + {\text{AS}}_{{2}} \;\left( {\text{s}} \right) + {\text{6H}}\left( {\text{l}} \right) \to {\text{C}}_{{2}} {\text{ASH}}_{{8}} \;\left( {\text{s}} \right) + {\text{C}} - {\text{S}} - {\text{H}}\;\left( {\text{s}} \right)$$

When the temperature was increased from 500 to 600 °C, the background hump between 30° and 40° became obvious, indicating that new crystalline phases formed and the crystallinity increased, which was caused by the transformation of the gel to a crystalline state at high temperature^47^. In the same range, the characteristic peaks of the particles prepared at 800 °C corresponded to Ca_2_SiO_4_, Na_2_Si_3_O_7_ and Ca_12_Al_14_O_33_. This result indicates that the pre-product was a silicate gel containing Ca and Na, the crystallinity increased between 600 and 700 °C, and the crystalline phase was formed at 800 °C. Therefore, the crystallization of silicate gel decreases the binding force between bentonite and carbide slag and increases the loss rate of particles. In the particles prepared at 600 °C, the characteristic peaks of Ca(OH)_2_ at 17.82° and 33.97° became flat and wide. The characteristic peaks of CaO were also found, which was due to the dehydration of Ca(OH)_2_ to CaO. This finding was consistent with previous conclusions^48,49^.

**Figure 9:**
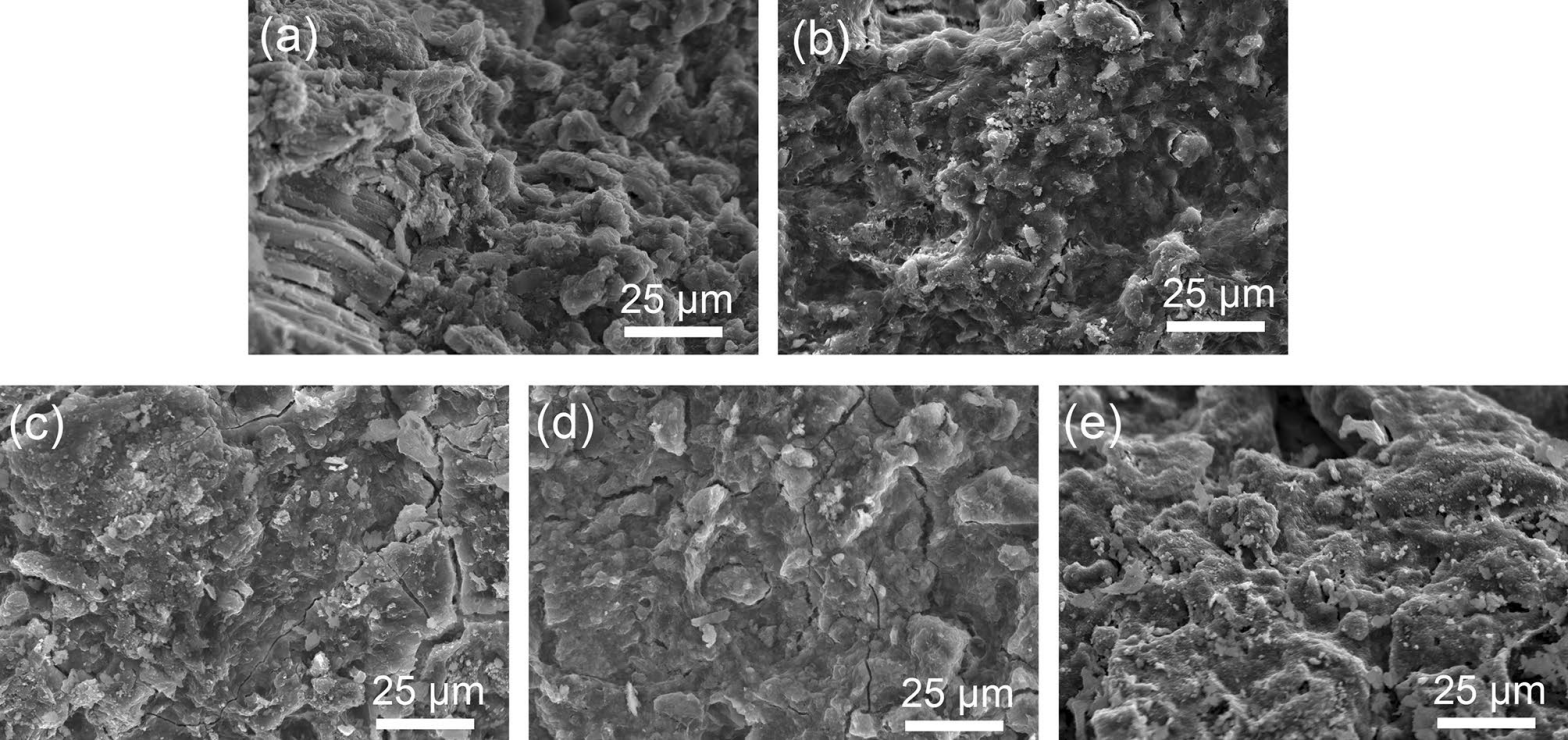
SEM images of composite particles at different calcination temperatures: (**a**) 25 °C, (**b**) 200 °C, (**c**) 500 °C, (**d**) 600 °C and (**e**) 800 °C.

Figure 9 shows SEM images of the surface morphologies of particles prepared at different temperatures. As shown in Fig. 9a, the surface morphologies of the uncalcined particles are rough, and the layered structure is clearly visible. Due to the evaporation of free water inside the particles calcined at 200 °C, small cracks appeared, and the layered structure became blurred (Fig. 9b). This is consistent with the XRD results. Regarding the particles calcined at 500 °C, the surface morphology became smooth, and the layered structure disappeared. The condensation of hydration products clarified the edges of cracks. Notably, this structure can combine bentonite and carbide slag closely^50^. Since Ca(OH)_2_ in the particles calcined at 600 °C was dehydrated and converted to CaO, the surface morphology became smoother (Fig. 9d). However, when the calcination temperature was increased to 800 °C (Fig. 9e), many irregularly distributed silicate crystals appeared, presenting complex particles surface morphologies. The crystals filled the original pores and blurred the edges of the surface cracks. A rough surface is not conducive to resisting the flow shear force and friction between particles, which is one of the reasons for the increase in the loss rate. However, an increase in crystallization is not conducive to the removal of Cu^2+^ and leads to a continuous decrease in the Cu^2+^ removal rate.

### Analysis of the sustained-alkalinity-release property

**Figure 10:**
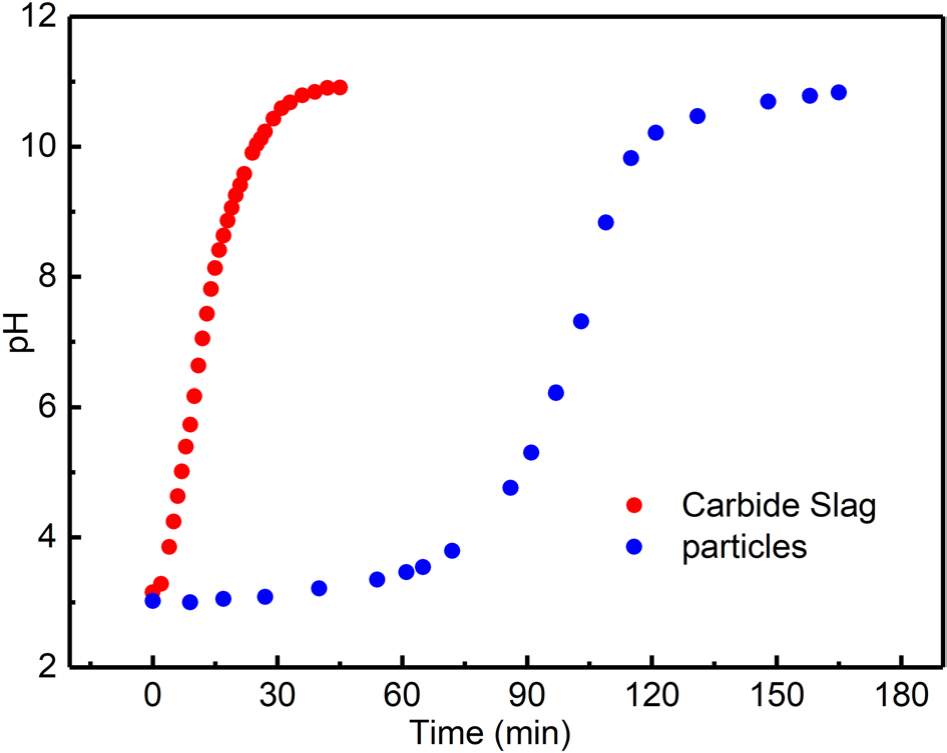
Alkalinity release rates of optimal composite particles and carbide slag powder.

Based on the above experiments, the mass ratio of bentonite to carbide slag was set as 3:7, the dose of Na_2_CO_3_ was set as 10 wt% of the total mass, the particles were calcined at 500 °C for 1 h. The prepared particles had the best mechanical strength and Cu^2+^ removal rate. The sustained-alkalinity-release properties of the particles were analysed. Figure 10 shows the time to achieve pH balance for 200 mg particles (containing 126 mg of carbide slag) and 126 mg carbide slag powder in solutions with initial pH = 3. The time for the release of alkalinity from the optimal particles to reach equilibrium was 180 min, four times that for carbide slag powder (45 min). Therefore, the composite particles demonstrated a good slow-release effect of alkalinity in water.

The (001), (101) and (110) crystal planes of Ca(OH)_2_ in the raw carbide slag powder, uncalcined particles, and calcined particles at 200 °C and 500 °C were fitted by XRD, and the crystallite size was calculated with the Scherrer Equation. The effect of calcination temperature on the crystallite size of Ca(OH)_2_ was compared and analysed. The fitting results are shown in Table 2.

**Table 2:** Fitting results for crystallite size of Ca(OH)_2_ in 3:7 particles with 10 wt% Na_2_CO_3_.

Crystal planes	(001)		(101)		(110)		Fit size (nm)
	FWHM	XS (nm)	FWHM	XS (nm)	FWHM	XS (nm)	
Carbide slag	0.309	275	0.347	250	0.298	313	267
25 °C	0.303	281	0.340	256	0.325	284	268
200 °C	0.321	363	0.370	233	0.320	289	249
500 °C	0.361	232	0.439	194	0.389	234	211

*FWHM* full width at half maximum, *XS* crystallite size.

Table 2 shows that the fit size of Ca(OH)_2_ in the carbide slag powder was 267 nm. After mixing with bentonite and Na_2_CO_3_ in the optimal proportion, the fit size of Ca(OH)_2_ in the particles without calcination was 268 nm, and the fit size exhibited little change. After calcination, the fit size of Ca(OH)_2_ decreased from 268 to 211 nm. This indicates that calcination effectively reduces the size of Ca(OH)_2_ crystallites. This decrease in crystallite size causes amorphous gel to encapsulate carbide slag better, thus buffering the alkalinity release of the particles.

## Conclusion

This study is the first attempt to use carbide slag, bentonite and a small amount of Na_2_CO_3_ to prepare sustained-alkalinity-release particles, namely, carbide slag composite sustained-alkalinity-release particles. The results show that the adhesion of the particles comes from the adhesion of bentonite itself and the formation of hydrated silicate gel. Na_2_CO_3_ enhances the activity of montmorillonite and silica in bentonite and plays a significant role in promoting the formation of the gel phase. The gel formed between the interface of the carbide slag and bentonite makes the two bonds. At room temperature, SiO_2_ is preferentially involved in the formation of gels. As the temperature increases, the reactivity of Si and Al in the montmorillonite structure increases to form hydrated silicate in amorphous form. Furthermore, the gel loses water and shrinks at high temperature, condensing the particle structure and obtaining good mechanical strength. However, at the temperatures above 600℃, the gel gradually transforms to the crystalline state, and the particle loss rate increases. The optimum preparation conditions of the composite sustained-alkalinity-release particles were as follows: bentonite to carbide slag mass ratio of 3:7, Na_2_CO_3_ dose of 10 wt%, and calcination temperature of 500 °C for 1 h. The fitting results for the crystallite size showed that the crystallite size of Ca(OH)_2_ decreased from 268 to 211 nm, which made the hydrated silicate gel encapsulate Ca(OH)_2_ better and reduced the alkalinity release rate of the carbide slag by 4 times. Therefore, carbide slag composite sustained-alkalinity-release particles can replace limestone as a new filling material in ALDs, which is beneficial for preventing the formation of AMD at the source.

## Supplementary Information

Supplementary Information.

## Data Availability

All data generated or analysed during this study are included in this published article (and its Supplementary Information files).
