# Supplementary figures and images for: Formation mechanism of carbide slag composite sustained-alkalinity-release particles for the source control of acid mine drainage
Source: Sci Rep. 2021 Dec 10;11:23793. doi: 10.1038/s41598-021-03277-w (PMC8664932; doi:10.1038/s41598-021-03277-w)

# Supplementary Figure S1


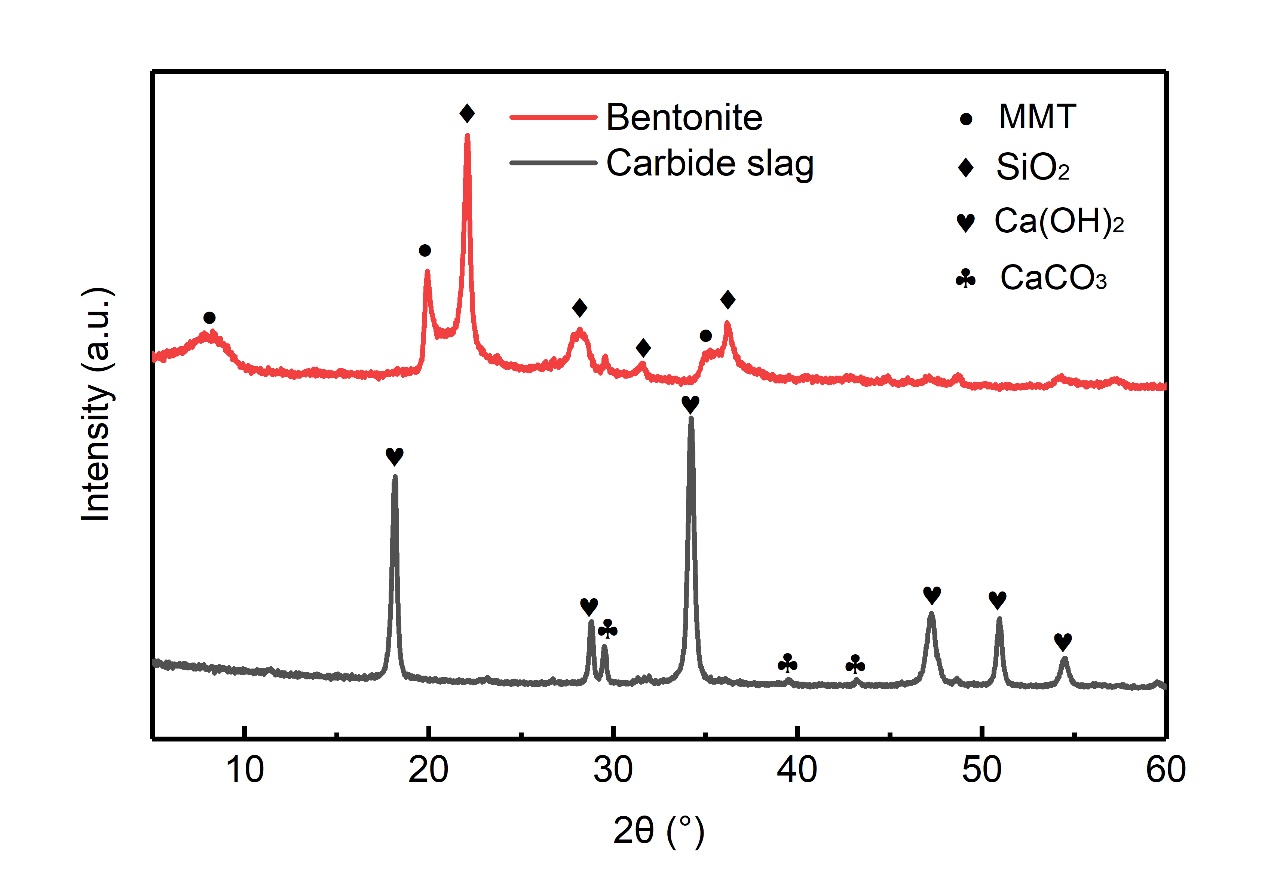


Fig. S1 XRD patterns of raw materials, MMT: montmorillonite.

Supplement: Supplementary file 1 — Supplementary Information. [file 41598_2021_3277_MOESM1_ESM.docx]
